# Prematurity and congenital malformations differ according to the type of pregestational diabetes

**DOI:** 10.1186/s12884-024-06470-7

**Published:** 2024-05-02

**Authors:** Monica Ballesteros, A Guarque, M Ingles, N Vilanova, M Lopez, L Martin, M Jane, L Puerto, M Martinez, M De la Flor, J Vendrell, A Megia

**Affiliations:** 1 Department of Medicine and Surgery, Rovira i Virgili University, Tarragona, Spain; 2 CIBER de Diabetes y Enfermedades Metabólicas Asociadas (CIBERDEM), Carlos III Health Institute, Madrid, Spain; 3 Department of Obstetrics and Gynecology, University Hospital of Tarragona Joan XXIII, Institut d’Investigació Sanitària Pere Virgili (IISPV), Tarragona, Spain; 4 Departament of Endocrinology and Nutrition, Research Unit, University Hospital of Tarragona Joan XXIII, Institut d’Investigació Sanitària Pere Virgili (IISPV), Tarragona, Spain; 5 Present Address: Institut d’Investigació Sanitària Pere Virgili (IISPV), Tarragona, Spain

**Keywords:** Pregnancy, Diabetes, Preterm delivery, High-risk pregnancy, Maternal-fetal medicine, Obesity

## Abstract

**Background:**

Diabetes mellitus (DM) is the most common metabolic disorder in pregnancy. Women with Type 2 DM seems to have no better perinatal outcomes than those with Type 1 DM.

**Methods:**

Single-center prospective cohort observational study. Pregnant women with diabetes (141 with Type 1 DM and 124 with Type 2 DM) that were followed in the university hospital between 2009 and 2021 were included in this study. Clinical data and obstetric and perinatal outcomes were collected.

**Results:**

As expected, women with Type 1 DM were younger and had a longer duration of diabetes than women with Type 2 DM. Obesity and chronic hypertension were higher in the group of women with Type 2 DM and their value of HbA1c in the second and third trimesters were lower than in Type 1 DM. No differences in prematurity were found, but more extreme prematurity was observed in Type 2 DM, as well as a higher rate of congenital malformations. The frequency of hypoglycemia and the weight of the newborn was higher in Type 1 DM. The maternal independent factors related to the weight of the newborn were: the glycemic control at the third trimester, the weight gain during pregnancy, and pregestational BMI.

**Conclusions:**

Newborns born to mothers with Type 1 DM were larger and had a higher frequency of hypoglycemia, while congenital malformations and precocious preterm was more associated to Type 2 DM. Metabolic control, weight gain and pregestational weight were important determinants of both obstetric and neonatal complications.

## Introduction

Pre-pregnancy diabetes mellitus (PDM) is associated with an increased risk of maternal and perinatal complications. It is estimated to affect 1% of pregnancies, but its prevalence is increasing progressively [1–4].

Previous evidence, predominantly drawn from retrospective studies, systematic reviews, and population-based analyses, has delineated disparities in pregnancy outcomes between Type 1 and Type 2 diabetes mellitus (DM). Some studies, such as Murphy et al. [5], have reported a higher frequency of prematurity, neonatal hypoglycemia, and large-for-age infants in Type 1 diabetes compared to Type 2 DM, while other studies have found no significant differences [6].

As we know from previous studies, when comparing pregnant women with Type 1 DM, at the time of pregnancy, those with Type 2 DM had a lower duration of diabetes and lower rates of diabetic complications. However, they had no better perinatal outcomes than those with Type 1 DM. It has been proposed that the presence of a greater number of cardiovascular risk factors at the beginning of pregnancy in women with Type 2 DM could be responsible for this worse prognosis [6, 7]. These prognoses are exacerbated by low rates of pregestational control [8] often associated with social deprivation and ethnicity [6].

In relation to weight gain during pregnancy, Type 2 DM women have lower weight gain during pregnancy, but still have a higher weight at the end of gestation, therefore, Type 2 DM women have worsening obesity and hypertension throughout gestation [9], and the creation of specially designed treatment measures would be necessary to improve these outcomes.

This prospective cohort study aims to compare obstetric and perinatal outcomes between women with Type 1 and Type 2 DM. Furthermore, as part of a prospective cohort investigation, the study seeks to examine modifiable factors contributing to these differences, including glycemic control, maternal obesity, and gestational weight gain. By conducting a comprehensive analysis, the research aims not only to provide insights for clinical practice but also to stimulate the creation of customized interventions aimed at improving pregnancy outcomes for women with pregestational diabetes.

## Methods

### Study design

This is a prospective observational cohort study conducted in a tertiary center, in which we analyzed obstetric and perinatal outcomes of a cohort of pregnant women with Type 1 and Type 2 DM monitored between 2009 and 2021 at the Joan XXIII University Hospital of Tarragona. Pregnancy information, including outcome and any maternal or fetal complications, was obtained prospectively from the patients and medical records since 2012, and incorporated in a diabetes and pregnancy database. All these women signed an informed consent form authorizing the use of the data for research purposes. The information corresponding to gestations occurring before 2012 was collected retrospectively from the electronic health records and incorporated into the database in a pseudonymized manner.

Pregnant women were monitored at the center’s multidisciplinary diabetes and pregnancy unit according to standard clinical practice, based on the recommendations of the Spanish Diabetes and Pregnancy Group (GEDE) [10, 11]. We included pregnant women with a single pregnancy with Type 1 or Type 2 DM that was diagnosed at least 6 months before the onset of gestation and who had been monitored in our center during pregnancy. We excluded women with gestational diabetes mellitus, multiple pregnancies, overt diabetes, those who terminated gestation in another center and those who terminated gestation at our center, but who had been monitored in another center.

During the first visit, a detailed medical history was extracted for each patient, with particular attention to the previous obstetric history (previous miscarriages and births, and age at pregnancy among others), duration and status of diabetes, related chronic complications (retinopathy, nephropathy), and arterial hypertension.

Pregnancy was defined as a reported or documented positive urine pregnancy test. We defined gestational age according to the estimated date of delivery based on ultrasound assessment at approximately 12 weeks’ gestation. Multiparity was defined as parity of > 2 deliveries. Pregestational BMI was calculated according to the following formula: weight at the first visit in kg over height in m^2^. Maternal BMI was grouped as underweight (< 18.5 kg/m^2^), normal weight (18.5–25 kg/m²), overweight (25–29.9 kg/m²), and obese ( > = 30 kg/m²). Gestational weight gain (GWG) was defined as the difference between the weight at the last antenatal visit and the weight at first antenatal visit, and was classified according to the 2009 Institute of Medicine (IOM) guidelines [12] as follows. GWG of 12.5–18 kg for underweight women; 11.5–16 kg for normal-weight women; 7–11 kg for overweight women; and 5–9 kg for obese women [13]. Preconception care was defined as any strategy aimed at optimizing the mother’s health during this period (such as optimizing glycemic control, folic acid supplementation or discontinuation of teratogenic drugs.

### Maternal and fetal outcomes

Miscarriage was defined as pregnancy loss at ≤ 22 weeks, and stillbirth defined as pregnancy loss after 22 weeks’ gestation in which the infant was not live-born. Congenital malformations (all prevalent, defined by ICD-10 codes). Chronic hypertension was defined as systolic blood pressure more than 140 mmHg and or diastolic blood pressure more than 90 mmHg or if a patient was taking antihypertensive drugs before pregnancy. Pregnancy-induced hypertension was diagnosed for systolic blood pressure values > 140 mmHg and diastolic > 90 mmHg after the 20th week of gestation and preeclampsia was defined as an elevation in blood pressure (according to the definition of gestational hypertension) together with proteinuria (300 mg of protein or more in a 24-hour urine collection or a result of 2 + or greater on a dipstick test when a 24-hour collection was not available), after 20 weeks’ gestation or other signs of organ damage. Microalbuminuria was defined as a urine albumin-to-creatinine ratio of > 30 mg/g on two occasions prior to the pregnancy. Macroalbuminuria was defined as a urine albumin-to-creatinine ratio of > 300 mg/g prior to pregnancy. Renal function was considered normal when estimated glomerular filtration rate was greater than or equal to 60. The presence of any type of retinal involvement related to diabetes was considered diabetic retinopathy.

Obstetric complications included vaginal tears, hemorrhage, uterine atony, uterine rupture and postpartum hysterectomy. Preterm birth was defined as completion of birth before the 37th week. We divided prematurity into Precocious (less than 35 weeks) and Late preterm (35–36 weeks). Birth weight classification was described as small for gestational age or SGA (< 10th percentile), appropriate for gestational age or AGA (10–90th percentiles), and large for gestational age or LGA (> 90th percentile), adjusted for infant sex and gestational age [14]. Neonatal hypoglycemia was defined as blood glucose levels lower than or equal to 45 mg/dL during the first 24 h of life [15]. Neonatal hyperbilirubinemia was defined as the presence of neonatal jaundice requiring phototherapy. Birth trauma was defined as brachial plexus palsy or clavicular, humeral, or skull fracturing. A composite variable of perinatal complications including neonatal hypoglycemia, hyperbilirubinemia requiring phototherapy, respiratory distress, admission to hospital intensive care unit (ICU) or intermediate care was also developed. HbA1C reflects average plasma glucose over the previous eight to twelve weeks. HbA1C was performed every four to six weeks, to monitor diabetes control and the mean for each gestational trimester was calculated (first trimester < 14 weeks, second trimester 14–26 weeks and third trimester > 26 weeks). Blood for HbA1c was collected in ethylenediaminetetraacetic acid vials and determined by HPLC (ADAMS-A1c HA-8180; Menarini Diagnostics, Firenze, Italy).

### Statistical methods

The statistical analysis using descriptive and inferential techniques was carried out using the SPSS v25 programme (IBM, Armonk, NY). Normality was assessed by the Kolmogorov Smirnov test. Quantitative variables were shown as mean and standard desviation while categorical variables were described as frequencies and percentages. The comparison among quantitative variables was analyzed by using the Student’s t-test and among categorical variables was studied by means of contingency table analysis and the Chi-square test. Logistic regression analysis was used to assessed the association between type of diabetes and some of the clinical outcomes that have shown some association in the univariate analysis. We chose those variables with a *p*-value < 0.20. We developed two models, in the first model we assessed perinatal and obstetrical outcomes, so we included as potential independent variables pregestational BMI, GWG and HbA1C in the third trimester along with the type of diabetes. In the second model, we assessed the association of congenital malformations, so we included pregestational BMI, maternal age, pregestational HbA1C along with the type of diabetes. The accepted level of statistical significance was *p* < 0.05.

## Results

### Maternal characteristics

Pregnant women with Type 1 DM were younger and had a longer duration of diabetes. They were more frequently nulliparous and the percentage of women who had some type of retinopathy was higher. None of the patients had renal failure or clinical polyneuropathy at the beginning of gestation. Women with Type 2 DM were more obese, although they gained less weight during gestation, but still have a higher weight at the end of gestation than Type 1 DM, therefore, Type 2 DM had more severe obesity, and had a higher percentage of hypertension at the beginning of gestation (7.3% vs. 1.4%; *p*-value: 0.017) (Table 1).

**Table 1:** Clinical characteristics of the population studied according to the type of DM

	Type 1 DM	Type 2 DM	*p* value
Maternal age (years)	31.8 ± 4.6	34.94 ± 5.2	0.001
Nulliparity (%)	50.4	22.6	0.001
Duration of diabetes (years)	14.2 ± 8.2	4.5 ± 5.5	< 0.001
Pregestational BMI (kg/m^2^)	23.9 ± 3.6	31.5 ± 7.1	< 0.001
. Underweight (%)	2.2	0.8	
. Normal weight (%)	63.8	17.8	< 0.001
. Overweight (%)	29	30.5	
. Obesity (%)	5.1	50.8	
Gestational Weight gain (kg)	13.9 ± 5.4	9.1 ± 6.1	< 0.001
Adequacy of weight gain			
. Insufficient weight gain n (%)	26 (22.2)	32 (30.2)	
. Appropriate weight gain n (%)	31 (26.5)	35 (33)	0.091
. Excessive weight gain n (%)	61 (51.3)	39 (36.8)	
Pregestational arterial hypertension n (%)	2 (1.4)	9 (7.3)	0.017
Retinopathy (%)	15.2	1.6	0.002
A1C (%, mean ± SD)			
. Pregestational	7.1 ± 1.3	7.2 ± 1.7	0.542
. 1st trimester	6.6 ± 1.1	6.6 ± 1.3	0.917
. 2nd trimester	5.9 ± 0.6	5.7 ± 0.6	0.011
. 3rd trimester	6.1 ± 0.6	5.8 ± 0.7	0.002
Prepregnancy care (%)	36.4	11.3	< 0.001
Ethnicity/Race (%)			
. European Mediterranean	89.2	46	
. North African	4.3	34.7	< 0.001
. Others	6.5	19.3	
Previous miscarriage (%)	30.85	41.1	0.074

Regarding the adequacy of weight gain throughout pregnancy and type of diabetes, no significant differences were observed between the two groups, although the highest percentage of women with both Type 1 and Type 2 DM was found in the group with excessive weight gain (Table 1).

The degree of glycemic control estimated by HbA1c levels before the start of gestation and in first trimester were similar in two groups, but in the second and third trimesters of gestation was found to be better in the group of women with Type 2 DM.

As far as ethnicity is concerned, the predominant ethnic group in both populations were European Mediterranean (69.9%), followed by the North African group, which accounted for more than one-third of the women in the group with Type 2 DM. The other ethnic groups, Hispanic, Asian and Black, were in the minority, which accounted for more than one-fifth of the women in the group with Type 2 DM and represented less than 7% of the group with Type 1 DM (Table 1).

Finally, the low rates of pregestational control should be highlighted. The overall percentage of the population that performs pre-conception control was 23.8%, being higher in women with Type 1 DM compare to women with Type 2 DM (36.4 vs. 11.3%; *p*-value < 0.001) (Table 1).

### Obstetrics outcomes

The overall percentage of obstetric complications was 13.8%. No differences were observed in obstetric trauma and obstetric outcomes except for the gestational week in preterm delivery. Neither no significative onset of hypertension during gestation between two groups was found, possibly because of insufficient cases.

No differences were observed in the prematurity rate between Type 1 DM and Type 2 DM groups. However, when preterm deliveries were divided in two groups, late and precocious, more than 60% of the preterm deliveries in the group of Type 2 DM were precocious, whereas all preterm deliveries of Type 1 DM were late preterm. No other significant differences were observed between the two groups (Table 2).

**Table 2:** Obstetric and perinatal outcomes according to the type of DM

	Type 1 DM	Type 2 DM	*p* value
**Obstetrics outcomes**			
Gestational week at delivery (weeks)	37.78 ± 1.02	37.31 ± 3.13	0.124
Induction of labour n (%)	100 (84.2)	88 (83)	0.838
Vaginal delivery n (%)	60 (50.4)	55 (50)	0.733
Gestational hypertension n (%)	1 (0.7)	5 (4)	0.070
Preeclampsia n (%)	6 (4.3)	6 (4.8)	0.820
Prematurity rate (%)	9.1	10.2	0.750
. Prematurity (weeks)	35.61 ± 0.50	31.50 ± 4.9	0.006
. Late prematurity n (%)	13 (100)	5 (35.7)	< 0.001
. Precocious prematurity n (%)	0 (0)	9 (64.3)	< 0.001
Obstetrical Complications n (%)	16 (13.9)	14 (13.6)	0.945
Obstetrical trauma n (%)	1 (0.9)	2 (2)	0.497
Miscarriage n (%)	17 (12.1)	9 (7.3)	0.190
Stillbirth (weeks)	37	27.66 ± 7.37	0.387
Stillbirth n (%)	1 (0.8)	3 (2.6)	0.354
**Perinatal outcomes**			
Female Sex n (%)	58 (48.7)	46 (44.2)	0.293
Birth weight (g)	3408 ± 505	3137 ± 817	0.003
Birth height (cm)	49.29 ± 2.28	48.61 ± 2.68	0.049
Adiposity Index	28.42 ± 4.76	28.04 ± 2.91	0.578
Macrosomia (≥ 4000g)	18 (15.3)	11 (10.4)	0.278
LGA (%)	45.3	36.1	0.173
SGA (%)	3.4	6.2	0.340
Apgar 1’	8.70 ± 1.11	8.51 ± 1.79	0.092
pH arterial	7.22 ± 0.12	7.23 ± 0.09	0.543
pH venous	7.25 ± 0.08	7.30 ± 0.09	0.002
Neonatal Complications n (%)	46 (39.7)	31 (31)	0.185
Hypoglycaemia n (%)	28 (24.6)	9 (8.9)	0.002
Respiratory distress Syndrome (RDS) n (%)	8 (7.1)	7 (7.1)	0.998
Hospital admission n (%)	25 (21.5)	19 (19.4)	0.148
Congenital Malformation n (%)	4 (3.5)	11 (10.8)	0.034

LGA: large for gestational age; SGA: small for gestational age; Neonatal Complications Composite variable (neonatal hypoglycemia, hyperbilirubinemia requiring phototherapy, respiratory distress, admission to hospital intensive care unit (ICU) or intermediate care)

### Perinatal outcomes

The children born to women with Type 1 DM were larger than those born to women with Type 2 DM, but there were no differences in the percentage of macrosomia, neonatal adiposity index, or when they were classified as LGA, SGA or AGA according to their weight, adjusted for sex and gestational age.

We found no differences between neonatal complications in isolation or as a composite variable with type of diabetes, only with neonatal hypoglycemia, was more frequent in children from mothers with Type 1 DM than in the group with Type 2 DM (Table 2).

Finally, the frequency of congenital malformations was higher in the Type 2 DM group than in the Type 1 DM (Table 2). The predominant malformations in our sample involved the heart, central nervous system, and the kidney, such as ductus arteriosus, ventricular septal defect (VSD), transposition of the great vessels, agenesis of the corpus callosum, microcephaly, polycystic kidney disease, renal duplicity, and hydronephrosis among others.

### Assessment of the influence of maternal clinical characteristics on the association between the type of diabetes and obstetric and perinatal outcomes

Next, we evaluated how weight gain, obesity at the beginning of gestation and the degree of metabolic control influenced the observed relationship between the type of diabetes and some clinical outcomes. We analyzed those that had an association with the type of diabetes with a *p*-value < 0.2.

For this purpose, we performed two models of logistic regression analysis. In the first model, LGA, neonatal hypoglycemia and the composite outcome of neonatal complications were included as dependent variables, whereas the type of diabetes, pregestational BMI, GWG, and third trimester HbA1C were independent variables.

After adjustment, the type of diabetes remained associated with neonatal hypoglycemia. Metabolic control was independently associated with an increase of all the clinical outcomes analyzed. Gestational weight was associated with an increased odds ratio of neonatal complications and LGA, whereas pregestational BMI was only associated with LGA. See Table 3.

**Table 3:** Adjusted logistic odds ratios for LGA, neonatal complications and neonatal hypoglycaemia

	Type of DM	Metabolic Control (Third trimester HbA1C)	Weigh Gain	Pregestational BMI
**LGA**				
. Odds Ratio	0.682	2.772	1.102	1.112
. 95% CI	(0.303–1.536)	(1.553–4.947)	(1.036–1.172)	(1.040–1.190)
. *p*-value	0.356	0.001	0.002	0.002
**Neonatal Complications Composite variable**				
. Odds Ratio	0.704	1.942	1.065	1.05
. 95% CI	(0.320–1.551)	(1.165–3.237)	(1.006–1.128)	(0.994–1.110)
. *p*-value	0.384	0.011	0.032	0.083
**Neonatal Hypoglycemia**				
. Odds Ratio	0.271	1.902	1.065	1.048
. 95% CI	(0.089–0.827)	(1.056–3.429)	(0.993–1.143)	(0.980–1.120)
. *p*-value	0.022	0.032	0.077	0.169

HbA1C: glycated hemoglobin, BMI: body mass index, LGA: large for gestational age; Neonatal Complications Composite variable (neonatal hypoglycemia, hyperbilirubinemia requiring phototherapy, respiratory distress, admission to hospital intensive care unit (ICU) or intermediate care)

In the second model, we assessed the association between the occurrence of malformations and the type of diabetes after adjusting for pregestational HbA1C, maternal age and pregestational BMI. The association between the type of diabetes and congenital malformations was lost and pregestational HbA1C was the only variable independently related to the occurrence of malformations (OR 1.682 (1.022–2.770); p:0.041).

## Discussion

In this study, involving pregnant women with Type 1 and Type 2 DM, followed according to standard clinical practice [10, 11], we demonstrate how, despite a similar degree of glycemic control at the beginning of gestation, the distribution of obstetric and perinatal complications varies depending on the type of diabetes. A relevant finding of our study is that early prematurity is higher among women with Type 2 DM, with no differences observed in the frequency of total preterm births between the two groups. We also highlight how potentially modifiable factors such as metabolic control, pregestational overweight, and excessive weight gain during gestation are associated with the onset of these complications.

Pre-gestational characteristics of the women included tend to be common in previous studies [6, 7, 15, 16]. But in contrast to previous studies [16], we did not observe differences in degree of glycemic control at the beginning of gestation between two groups. Most of the women included in our study were European Mediterranean, but among women with Type 2 DM there was a higher percentage of women from North African, unlike other studies which were of other ethnicities [16], were also related to greater socioeconomic deprivation, a higher rate of obesity, language difficulty, and reduced awareness of illness, which affect follow-up and possibly obstetric outcomes.

The available evidence strongly suggests that structured preconception care for women with pregestational diabetes reduces the risk of major congenital anomalies and perinatal mortality in women with Type 1 and Type 2 DM and is cost-effective [17]. In our study, we observed more preconception screening in Type 1 DM than in Type 2 DM (36.4% vs. 11.3%), which could be attributed to the fact that the latter are less aware of the disease and its impact on pregnancy; on the other hand, the overall percentage of the population that gets preconception screening was 24.7%, which is lower than in published studies [2], which reaches 40–60%, the creation of specially designed care devices would be necessary to improve these outcomes. This is even more important in women belonging to disadvantaged social groups.

Pregestational diabetes mellitus and maternal hyperglycemia, estimated by HbA1c, during the time of organogenesis (pregestational or first trimester) has been directly related to the occurrence of neonatal congenital malformations in population-based [17, 18, 20, 21]. Maternal hyperglycemia is a known teratogen with detrimental effects on the fetal cardiac, renal, musculoskeletal, and central nervous system [19]. We found a higher percentage of malformations in mothers with Type 2 DM than in mothers in Type 1 DM similar to previous studies [15], and different from others [22]. This could be explained by the fact that their group [22] of pregnant women with Type 2 DM has better pregestational glycaemic control and lower BMI. Although maternal obesity has been postulated as a mediating factor for a greater increase in neonatal congenital malformations [6, 15] in Type 2 DM, it did not reach statistical significance in our study and we only observed that pregestational glycaemic control was independently associated.

The mean at delivery was 37 weeks, in both types of DM, most often through the decision of the antenatal team to deliver by induction of labor or cesarean section when signs of maternal (glycemic control) or fetal complications developed. Information about preterm birth in pregnancies complicated by pregestational diabetes is conflicting. Recent studies [15] show preterm birth rates between 33 and 45% in women with Type 1 DM and around 20% in Type 2 DM. In contrast, our overall prematurity rates are lower, around 10%, and comparable to other pregnant women without diabetes, due to exhaustive metabolic and obstetric control protocols, in order to avoid iatrogenic prematurity and reduce neonatal morbidity. We found no difference in prematurity between the two types of DM, nor between prematurity and the degree of obesity. In contrast to our study, a recent national population-based cohort study found a higher rate of preterm delivery among women with Type 1 DM compared with Type 2 DM, could be explained by poorer glycaemic control at the end of gestation in Type 1 DM in this cohort [5].

In our study, when preterm deliveries were divided in two groups, late and precocious, more than 60% of the preterm deliveries in the group of Type 2 DM were precocious. The extreme prematurity, which is only found in Type 2 DM, must be associated with other factors such as obesity, pregestational weight, pregestational arterial hypertension … which differentiates them from Type 1 DM. These influences must be assumed as we do not have any value in Type 1 DM to be able to prove their implication. Our Type 1 DM have better previous metabolic control with a lower rate of nephropathy than in the studies [23] that could determine an earlier termination. In the same series, it was also observed that the mean age of preterm births in Type 2 DM was 34 weeks compared to 35 weeks in Type 1 DM, although this was not statistically significant. A larger sample could ratify these values, as an influential factor could be hypertensive disease during gestation, but given its low prevalence, we did not observe significant differences in both groups, although the percentage was higher in Type 2 DM than in Type 1 DM.

Obesity and excessive weight gain during pregnancy have been associated with increased risk of LGA children [15], probably through maternal and fetal dysregulation of glucose, insulin, lipid, and amino acid metabolism [24]. This agrees with our study in which obesity, GWG, and especially glycemic control in the third trimester were associated with LGA children, independent of the Type of DM. Balsells et al. [6] found rates of LGA similar between two types of diabetes, in contrast to other studies [25], possibly because Ladfors et al. [25] show a higher excessive weight gain in Type 1 DM than in Type 2 DM, in contrast to our series.                                                                                                                                                             No differences were observed in relation to the type and route of gestational termination, number of miscarriage and stillbirth. In contrast to Guarnotta et al. who found that women with type 2 DM showed higher prevalence of abortion than Type 1 DM [9]. A review of the data shows a higher percentage of stillbirth in Type 2 DM, all of them premature, but this does not appear to be significant, possibly due to the small sample size. The only stillbirth of Type 1 DM was at term.

In terms of obstetric complications, which included phenomena such as vaginal tears, hemorrhage, uterine atony, uterine rupture and postpartum hysterectomy, no significant differences were observed. The overall percentage of obstetric complications was 12.8%.

Neonatal hypoglycemia was more frequent in the children of mothers with Type 1 DM than in the group with Type 2 DM, and was influenced by metabolic control in the third trimester and not by maternal weight gain or pre-pregnancy weight. No other differences were observed in the perinatal complications between two types of diabetes, studied individually. When we considered them together, as a composite variable were associated to metabolic control in the third trimester and GWG, regardless of the type of diabetes.

Limitations of the study are the limited number of patients to test for low prevalence complications, no data on other risk factors such as smoking, drugs and other medications (like aspirin) that pregnant women are taking and a predominant ethnic group European Mediterranean. The strengths unlike others studies, our sample is a single-center study, mostly prospective study with a considerable number of patients and homogeneous sample comparing only pregnant women with Type 1 and Type 2 DM, without including gestational diabetes or control groups. The clinical applications would consist of improving the rate of preconception screening, with an emphasis on the control of risk factors (obesity, sedentary lifestyle, hypertension) with the aim of reducing the rate of extreme prematurity associated with significant neonatal morbidity. Further studies on the control of these variables are needed.

In summary, although there was no difference in the rate of prematurity between the two types of DM, Type 2 DM had a more extreme prematurity than Type 1 DM, and although we observed a greater presence of malformations in Type 2 DM it seems to be more mediated by pregestational metabolic control. The frequency of hypoglycemia and birth weight was higher in Type 1 DM. Other potentially modifiable pre-gestational factors such as pregestational BMI, maternal weight gain and glycemic control play an important role in the occurrence of complications, that could implications than o more important than type of diabetes must be in consideration, ongoing quality care for persons with diabetes is an important opportunity for prevention.

## Data Availability

The data that support the findings of this study are not openly available due to reasons of sensitivity and are available from the corresponding author upon reasonable request.

## Abbreviations

BMI: Body Mass Index
DM: Diabetes mellitus
GWG: Gestational weight gain
